# Dataset of Tactile Signatures of the Human Right Hand in Twenty-One Activities of Daily Living Using a High Spatial Resolution Pressure Sensor

**DOI:** 10.3390/s21082594

**Published:** 2021-04-07

**Authors:** Javier Cepriá-Bernal, Antonio Pérez-González

**Affiliations:** Department of Mechanical Engineering and Construction, Universitat Jaume I, 12071 Castellón de la Plana, Spain; jcepria@uji.es

**Keywords:** human grasping, tactile signatures in ADL, pressure sensor, dataset

## Abstract

Successful grasping with multi-fingered prosthetic or robotic hands remains a challenge to be solved for the effective use of these hands in unstructured environments. To this end, currently available tactile sensors need to improve their sensitivity, robustness, and spatial resolution, but a better knowledge of the distribution of contact forces in the human hand in grasping tasks is also necessary. The human tactile signatures can inform models for an efficient control of the artificial hands. In this study we present and analyze a dataset of tactile signatures of the human hand in twenty-one representative activities of daily living, obtained using a commercial high spatial resolution pressure sensor. The experiments were repeated for twenty-two subjects. The whole dataset includes more than one hundred million pressure data. The effect of the task and the subject on the grip force and the contribution to this grip force made by the different hand regions were analyzed. We also propose a method to effectively synchronize the measurements from different subjects and a method to represent the tactile signature of each task, highlighting the hand regions mainly involved in the task. The correlations between hand regions and between different tasks were also analyzed.

## 1. Introduction

The ability to grasp and manipulate is a distinctive feature of human beings. A better understanding of the dynamics of human grasping is essential in different fields, such as robotics, prosthetics, ergonomics, or rehabilitation.

Autonomous successful grasping with multi-fingered hands is one of the main challenges for spreading robotics in non-industrial applications [1,2,3]. Industrial robots manipulate very specific objects in controlled environments, but assistive robots or humanoid robots are called to work in unstructured environments and grasp a multiplicity of possible objects. Prosthetic hands must also be able to help disabled people in activities of daily living (ADL) and deal with a high variety of objects. Successful grasping requires sensing the environment and performing efficient arm and hand motions based on the sensory information, mainly visual and tactile. This process is learned by humans in early years. Grasping methods in robotics have approached the problem using mainly computer-vision as sensory input [4], even when dealing with previously unknown objects [5]. Machine learning has been applied increasingly in recent years for improving the robustness under changes in environment and illumination [6] or for dealing with multiple randomly oriented or piled up objects [7,8] but keeping vision as the main sensory input. However, as highlighted by Li et al. [9], the use of tactile information can improve the grasp stability in the execution stage, because it provides information related to important parameters for the feasibility of the grasp, such as the weight of the object or its center of mass. Tactile signatures produced by objects in contact with tactile sensors located in artificial hands can help grasping novel objects [10,11]. However, tactile sensors used in robots still have important limitations in terms of spatial resolution, geometrical adaptation to the hand, and robustness [12]. Additionally, there are no clear procedures to use efficiently the tactile information [13]. A considerable amount of research is still necessary to use efficiently tactile sensors in robotic grasping.

The impressive recent work by Sundaram et al. [14] proposes spreading the use of large datasets of tactile information with high spatial resolution coming from human manipulation for improving our understanding of human grasping. They suggest the use of machine learning tools to distil the high-level relationships coming from this tactile information in order to transfer this knowledge to artificial grasping in robots or prostheses. In that work, the authors presented a low-cost glove that incorporates 548 individual sensing elements or sensels, which cover the main areas of the hand. They used the glove to measure contact pressures while interacting with 26 different objects. The experiments were conducted with a single subject and consisted in touching the objects and lifting them for weight estimation. The results suggest that machine learning can be used to identify objects from the tactile signatures with reasonable classification accuracy.

Prior to the cited work by Sundaram et al. [14], most of the previous studies about grasping forces exerted by the human hand focused mainly on pinch prehension or used specifically instrumented devices rather than real objects [15,16,17], due to the difficulties associated with sensor location. Most of the studies centered on cylindrical objects or handles [18,19,20,21,22,23], because cylindrical objects can be easily wrapped with thin pressure sensors. The use of specially designed instrumented objects with specific locations for the sensors is an alternative [24], but this approach may prevent the spontaneous selection of the grasp by the subjects. Attention has been drawn to the lack of research on grip-force distribution in the hand during functional whole-hand grasping [25] and can be partially explained by the difficulty of finding suitable and non-invasive sensors. Pylatiuk et al. [25] analyzed functional manipulation of bottles using specially designed discrete force sensors placed on the hand. Hermsdörfer et al. [26] analyzed forces during the manipulation of several objects associated with routine activities of daily living, but they only measured contact forces on the fingertips of three grasping fingers. We also contributed with an analysis of the force sharing in two different manipulation tasks with bottles [27] using eight thin wearable sensors. Recently, Silva et al. [28] analyzed the differences in contact pressure distribution in the hands for two different wheelchair hand rim models using pressure sensors attached to gloves. Despite these advances, the force sharing among hand regions in functional tasks related to activities of daily living (ADL) has not been sufficiently studied.

The aim of the present study is to contribute to a better knowledge about how humans modulate the contact force exerted on the objects for successful manipulation in a representative set of ADL. In particular, we are interested in the magnitude of the grasping force necessary for these activities, in how it changes during the execution of the activities, and in how the grasping force is distributed among the different hand areas. As outlined above, similar analyses found in the literature lack the sufficient spatial resolution or do not cover a representative set of realistic ADL. We are also interested in analyzing if there are common patterns in the spatial distribution of the contact forces for different activities, which could be exploited by artificial hands for successful manipulation. Finally, we want to analyze to which extent the contact force and its spatial distribution is unique for a given task or, on the contrary, it is subject-specific.

With this objective, in the present study we contribute in the same direction proposed by Sundaram et al. [14] with the results of a set of experiments providing tactile information coming from 22 subjects performing 21 different ADL. The subjects wore a glove that incorporates a commercial high resolution pressure sensor from Tekscan, the Tekscan Grip System, which provides instantaneous pressure information in 361 different sensels. This open dataset [29] is, to our knowledge, the largest and most diverse database with tactile information of the human hand in ADL. Here, we include analyses of these results, looking for possible correlations between tasks or between sensing regions to highlight the main results. We also provide methods to transform the huge amount of spatiotemporal information provided by the experiments into useful information for understanding the grasping action in ADL. Further analyses of these data could also provide new insights to understand human grasping. We hope that the information contained in the dataset helps in the near future for the design or control of prosthetic or robotic hands.

## 2. Material and Methods

### 2.1. Subjects and Tasks

Twenty-two right-handed subjects (12 female and 10 male), aged 37 ± 8.2 (mean ± standard deviation), participated in the experiment. None of them had a history of trauma or pathology in the upper limb. Subjects gave informed consent to the experiment, whose protocol was approved by the Ethics Committee of the university and was in compliance with the Declaration of Helsinki. The mean hand length of the subjects, from the wrist to the tip of the middle finger, was 186.1 mm with a standard deviation of 11.4 mm and a range from 158 mm to 205 mm. The mean hand breadth, at the level of the metacarpal heads, was 81.7 mm with a standard deviation of 6.7 mm and a range from 72 mm to 95 mm.

**Figure 1:**
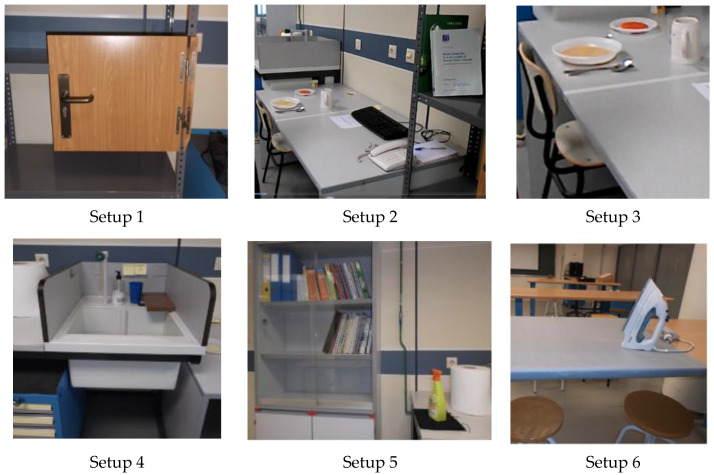
Setups arranged in the laboratory to perform the 21 selected activities of daily living (ADL).

**Figure 2:**
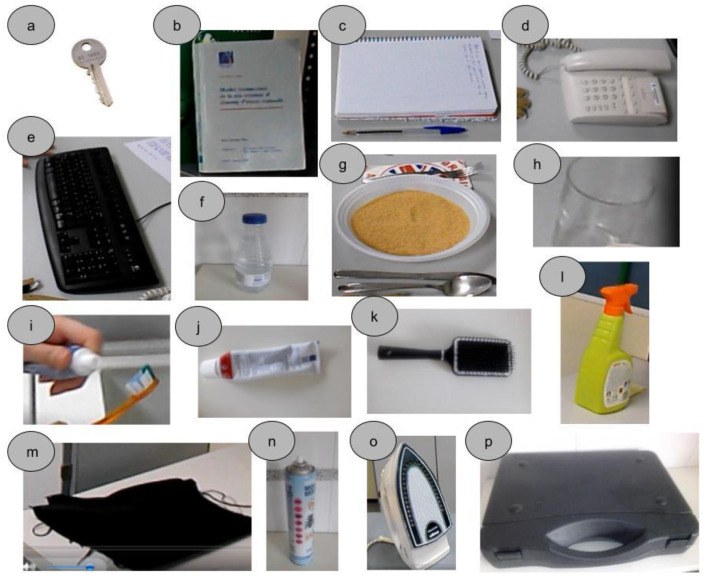
Objects used in the tasks: (**a**) key, (**b**) book, (**c**) pen, (**d**) phone, (**e**) keyboard, (**f**) bottle, (**g**) spoon, knife, fork, (**h**) glass, (**i**) toothbrush, (**j**) toothpaste tube, (**k**) comb, (**l**) pistol grip sprayer, (**m**) cleaning cloth, (**n**) aerosol can, (**o**) iron, (**p**) briefcase.

Each subject was instructed to perform the same 21 different tasks, using a variety of objects. The tasks corresponded to common ADL, selected to cover most of the areas related to the hand function of the different chapters of the International Classification of Functioning, Disability and Health (ICF). Figure 1 shows the six different setups arranged in our laboratory to perform the tasks, representing a common environment for these tasks in daily life. The objects used in the tasks are shown in Figure 2. Table 1 contains the main information about the tasks, objects manipulated and posture adopted by the subject during the task, and Table 2 contains a description of the sequence of actions performed. Each task was demonstrated to the subject immediately before the experiment and then they were asked to repeat it in the same way, at a natural speed. At the beginning of the task, the subjects adopted a relaxed neutral posture, either seated or standing, without initial contact of their hands with the objects. The objects were initially on a table, shelf, drawer, or ground and the subject’s hands were lying down in a relaxed position for standing posture or resting on the table for seated posture. For each task, at a signal of the experimenter, the subject grasped the object, performed the task, and returned to the neutral posture. Each subject performed each task once, all in the same session, and in the order indicated in the Table 1, with a brief resting period between them.

### 2.2. Instrumentation

**Figure 3:**
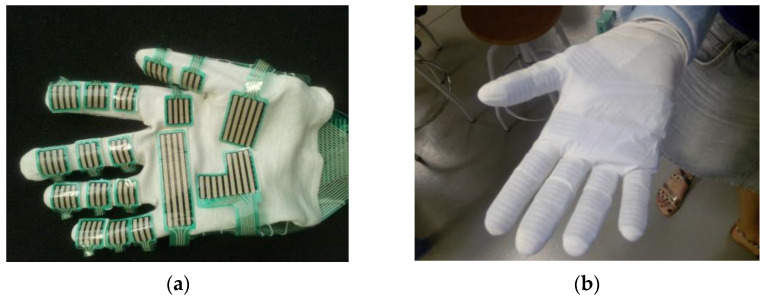
Pressure sensor sewn onto a cotton glove (**a**), final disposition in a subject’s hand covered with the additional polyethylene and latex gloves (**b**).

The subject’s right hand was instrumented with a thin resistive pressure sensor Grip 4256E, as part of the Grip System from Tekscan, Inc., Boston, MA, USA. This sensor contains 361 sensing elements or sensels, grouped in different sensing regions, covering the most important grasping contact areas of the volar side of the hand, as can be seen in Figure 3a. The pitch between sensel rows and columns in the sensing regions is 4 mm. To facilitate the placement of the sensor in the subject’s hand, it was sewn onto a cotton glove (Figure 3a), following the manufacturer instructions. For the experiment, the subject wore the cotton glove in his/her right hand, then it was covered by a second very thin, transparent, food-service polyethylene glove and finally by a thin disposable elastic latex glove (Figure 3b). The use of these additional gloves protected the sensor from direct contact with the objects and limited the change of position of the sensor with respect to the subject’s hand, helping also to obtain a friction coefficient with the objects more similar to that of the human hand. The sensor was connected to a hub attached to the subject’s arm, which sent the measurements to a computer through a USB connection.

The sensor was calibrated, equilibrated and tared to zero before the experiment, following the manufacturer instructions. We used the sensor map for a pressure range between 0 and 75 psi (0 to 517 kPa). During the task, the sensor recorded the pressure in each sensel at a sampling rate of 50 Hz. Taking into account the mean execution time per task, which was close to 14 s, the frequency of 50 Hz, 21 tasks, 22 subjects, and 361 sensels, the total amount of recorded information from the experiments was composed of 14 × 50 × 21 × 22 × 361 pressure values, more than 1.17 × 10^8^ numeric data.

### 2.3. Data Processing and Analysis

The pressure data registered were processed in Matlab^®^. The curves of recorded pressure versus time for each sensel were first filtered with a median filter with a window size of three, to eliminate noisy artifacts. The total contact force in the hand or grip force (GF) in a particular time instant was computed by summing up the forces corresponding to all the sensels. Saturated sensels were discarded, representing 2% of the registered data distributed among 215 different sensels across all the experiments. The force in a sensel was computed by multiplying the sensel pressure by the area associated to each sensel in the sensor matrix, 16 mm^2^.

#### 2.3.1. Synchronization of Measurements from Different Subjects

**Figure 4:**
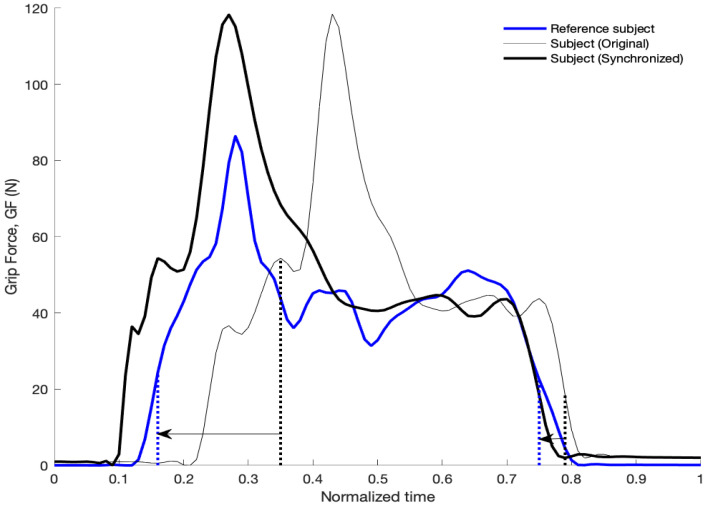
Time warping of the grip force (GF) curve corresponding to subject 1 for task 2, including the reference subject curve and Table 1. t1ref = 0.16, t2ref = 0.75, t1 = 0.35, t2 = 0.79.

As the subjects executed the tasks at their own pace, the curves of pressure or force versus time recorded in the experiment for the same task were not synchronized from subject to subject. Dynamic time warping (DTW) techniques can be applied for this alignment of time series. However, as other authors have reported [30] and we observed in our analyses, DTW can introduce potential singularities with excessive deformation of original signals and a loss of features of the original signals. Because of this, here we used a custom warping technique in order to improve the alignment of peaks and valleys in the signals without distorting the original signals. The procedure consists of three steps. In the first step, the recorded times during the task were normalized to range between 0 and 1 and then the GF curves were interpolated for relative time increments of 0.01. In the second step, the curves of GF were filtered with a zero-phase third order low-pass Butterworth filter with a cutoff frequency of 10Hz and averaged across subjects; for each task, the subject whose curve showed the lowest Euclidean distance with the averaged curve was taken as the reference subject for synchronization. In the third step, an optimal time warping was obtained for each subject in order to minimize the differences in the curve of GF versus time with respect to the reference subject. Four parameters were considered as variables for calculating the optimal time warping for a particular subject, corresponding to two time instants of the subject (t1, t2) and two time instants of the reference subject (t1ref, t2ref). The curve of the GF force for the subject was divided into three sections (0–t1, t1–t2, t2–1) and each segment was time-warped in order to move t1 to t1ref and t2 to t2ref. Figure 4 shows an example for task 2, including the reference–subject curve, the original subject curve, and the synchronized one. The Euclidean distance between the first derivatives of the subject curve and the reference curve was taken as the cost parameter for the optimization. The selection of the first derivative of the force instead of the force itself is explained by the better performance for synchronizing the peaks and valleys of the curves, observed in the preliminary tests performed. The time instants t1 and t1ref were bound to be within the range of normalized times 0.15–0.35 and the time instants t2 and t2ref to be within the range 0.65–0.85. This range for shifting the curves is enough to accommodate the interquartile range of task duration among the different subjects. The genetic algorithm “ga” included in the Global Optimization Toolbox of Matlab was used for the optimization. Other algorithms based on the gradient showed poorer results, due to the existence of local minima in the cost function.

Based on the results obtained for the synchronization of the GF, the curves of pressure versus time for each sensel were also interpolated to normalized time increments of 0.01 and shifted based on the same warping obtained for the corresponding task and subject.

#### 2.3.2. Analysis of Forces for the Different Tasks and Hand Regions

Once the recorded pressures were synchronized among the subjects, following the method described in the previous section, mean curves of the GF force during the different tasks were obtained by averaging among subjects. In order to avoid the effect of outliers, the four subjects whose curve of GF for a task presented the highest Euclidean distance with respect to the mean curve for this task, were discarded for subsequent analyses. The forces for each subject in a particular task were scaled, before averaging, in order to produce the same mean GF as that of the reference subject for this task. This scaling tried to compensate for differences in the level of force recorded among subjects, as a consequence of differences in sensor fitting from subject to subject. This allows reducing the variability, for a better understanding of the different tasks, and is equivalent to assume that the mean force required is mainly defined by the task.

**Figure 5:**
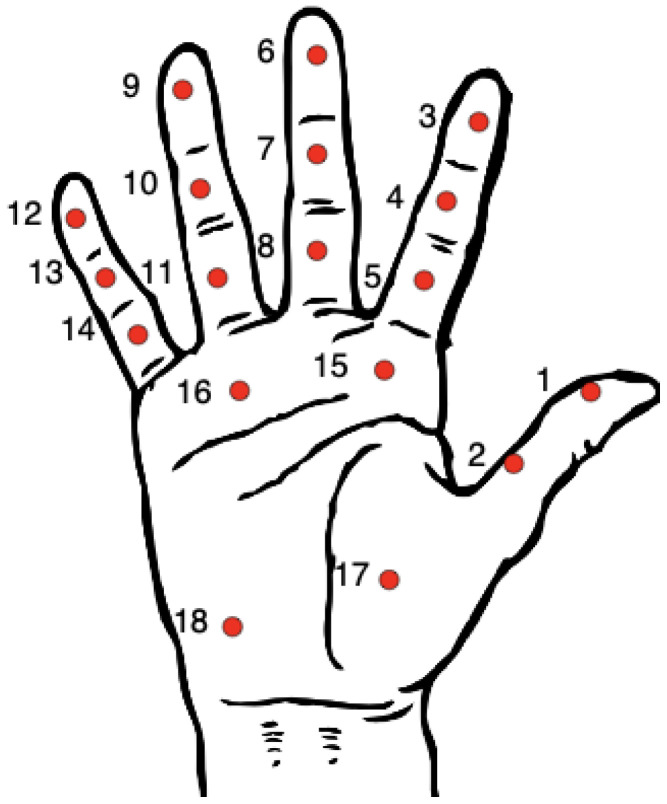
Sensor regions and their approximate locations in the hand palm.

The contribution of the hand areas to the GF was analyzed. To do this, the forces were computed separately in the 18 different sensing regions of the sensor (Figure 5), corresponding to different anatomical locations, as detailed in Table 3. The instantaneous contribution to the grip force (CGF) for each sensing region was defined as the ratio, for a time instant, between the force in this sensing region and the GF. The mean CGF for a task and subject was obtained as the mean value of CGF during the instants of the task in which the GF was higher than the average GF during the task for this subject. This procedure allows to consider only the part of the task in which the force level is significant for the task. The correlation among regions and among tasks was analyzed using Pearson’s linear correlation coefficients applied to the CGF values.

To understand the approximate mechanical requirements for the different contact regions, the peak values of force and pressure were obtained for each contact region and for each subject and then averaged among subjects for each task. The maximum values of these averages were obtained as indicators of the mechanical requirements for each hand region in ADL.

#### 2.3.3. Effect of Task and Subjects on the Results

To know if the task or the subject had a significant effect on the tactile signatures recorded, we performed two-way ANOVA analyses on the GF and on the CGF for each region, with “subject” and “task” as independent factors. Additionally, the possible effect of sex differences on the same results was investigated independently for each task, with an ANOVA test on the GF and a MANOVA test on the CGF. The dependent variable in all these statistical analyses was either the mean value of the GF or the CGF for each region. The mean value was obtained from the part of the task with a GF greater than the mean value during the whole task. The GF values used for these analyses did not include the scaling factor commented above for equalizing the mean GF among subjects.

## 3. Results

**Figure 6:**
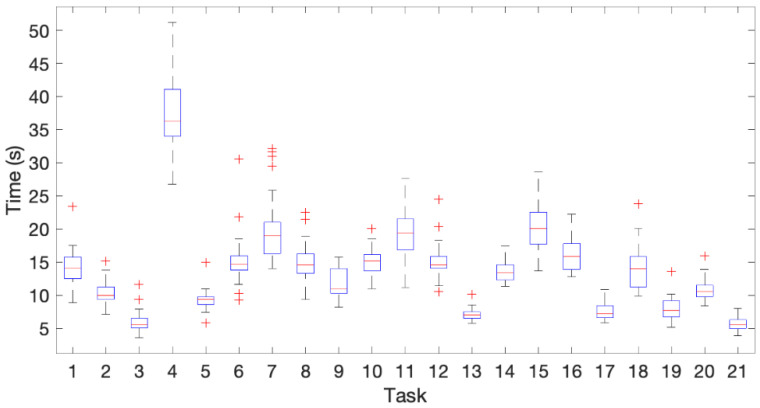
Boxplot of time spent in the execution of the different tasks.

Figure 6 shows a boxplot with the time spent for performing the different tasks. Task 4 was the slowest one and task 21 the fastest one on average. For some tasks, the time needed by most of the subjects was very similar (3, 5, 13, 14, 17, 20, 21). A greater variability in the time spent by different subjects was observed in precision tasks, such as 4 (hand writing), 7 (typing on a computer keyboard), 11 (cutting with a knife), 15 (serving toothpaste), 18 (cleaning a glass window with a cloth). It must be noted that the variability among subjects was influenced by the different reaction time of the subjects after the experimenter gave the signal to start the task. The number of outliers did not exceed four for any task.

**Figure 7:**
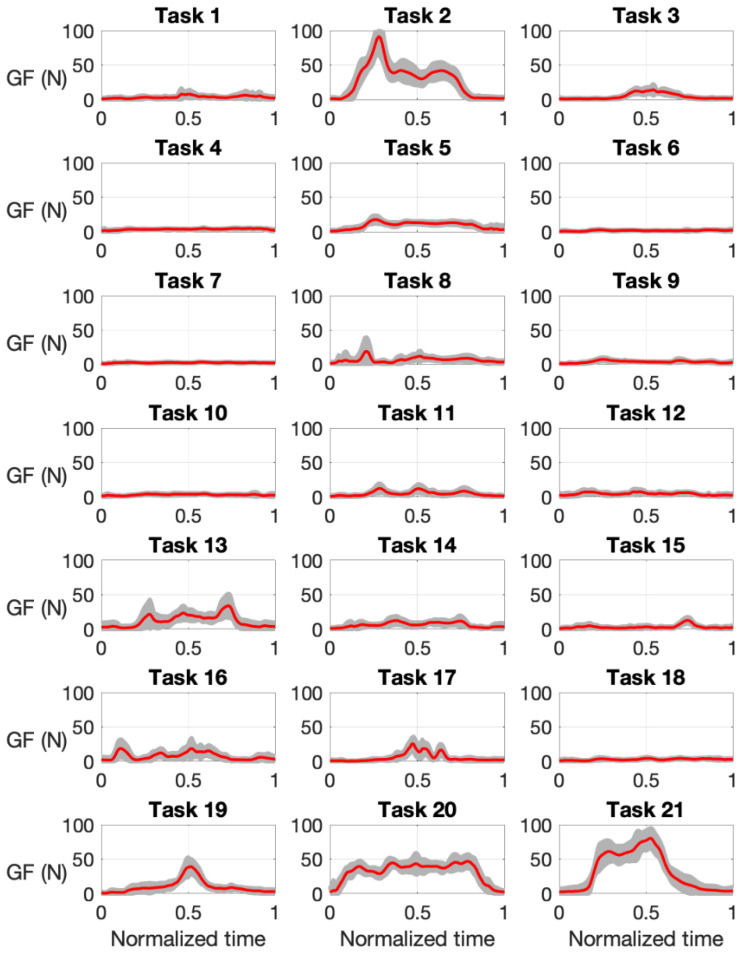
Mean grip force (GF, in N) versus normalized time for the different 21 tasks with curves for each subject scaled for a common mean GF in each task. Shaded areas show the standard deviation.

Figure 7 shows the mean time course of the GF for the different tasks, with the shaded area showing the standard deviation (*n* = 18). The tasks requiring the highest grasping force were task 2 (turning a handle to open a door) and task 21 (lifting a briefcase from ground level), with a peak GF close to 100 N, whereas other tasks required very low forces, such as 6 (dialing phone numbers) or 7 (typing on a computer keyboard). Intermediate peak forces close to 50 N were observed for tasks 13 (opening and closing a water tap), 19 (spraying insecticide), or 20 (ironing clothes).

**Figure 8:**
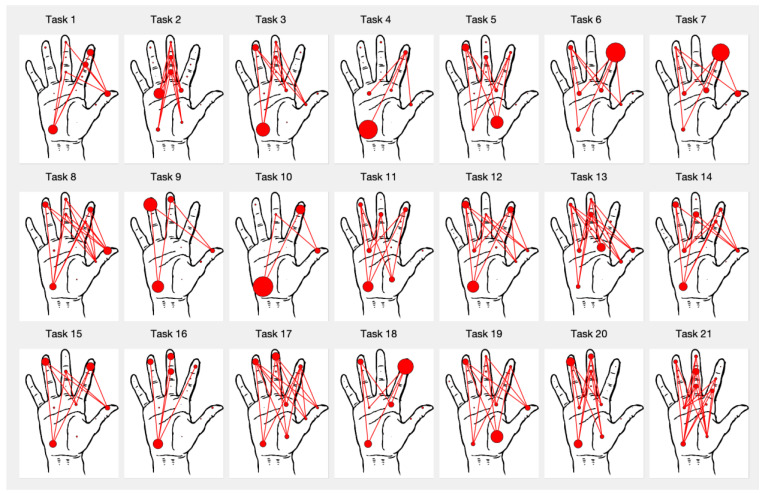
Characteristic pattern for each task in the mean contribution to grip force (CGF) for the different sensing regions, averaged among subjects. Circle sizes are proportional to the regions CGF and lines connect regions of the fingers with other regions of the thumb or palm, if CGF for both regions is greater than 5%.

Figure 8 shows a graphical tactile signature of the mean contribution of the different regions for each task, averaged among the subjects. The radii of the circles are proportional to the mean CGF of the corresponding region and the lines connect regions of the fingers with other regions of the thumb or palm when the mean CGF of the connected regions are both greater than 5%, providing an insight into the force opposition patterns for this task. Power tasks, such as 2, 13, 17, 19–21 show a greater number of regions recruited, in response to the higher forces required. The hypothenar eminence (region 18) shows a significant contribution in some tasks, providing the main force opposition to the fingers for the grasp (as in feeding tasks 9–12, or the book handling task 3) or acting as a support in contact with the table (task 4, writing with a pen). The thenar eminence (region 17) is a significant opposing region in tasks requiring cylindrical or diagonal power grasps, such as those appearing while grasping the phone handset (task 5) or spraying (tasks 17 and 19) and collaborates with the hypothenar eminence in others, such as ironing (task 20), cutting with a knife (task 11), or in hook grasp (task 21). The thumb is used as an opposing region in tasks requiring force and dexterity, such as turning a key (task 1), screwing or unscrewing (tasks 8 and 13), using the spoon or the fork (tasks 10, 12), or brushing the teeth (14, 15). It has also a significant contribution to the use of the computer keyboard (task 7). The distal palmar area is used mainly in power grasps, such as turning the door handle (task 2), grasping bottles (task 19), or other handles (tasks 20, 21), but also providing a resting support in keyboard hitting (task 7). With respect to the fingers, the contribution of the little finger is clearly negligible with respect to that of the index, middle, and ring fingers. Distal regions of the fingers have a greater mean CGF than the middle and proximal regions. The distal phalanx of the index finger is clearly dominant in tasks for pressing buttons or keys, such as 6 or 7.

**Figure 9:**
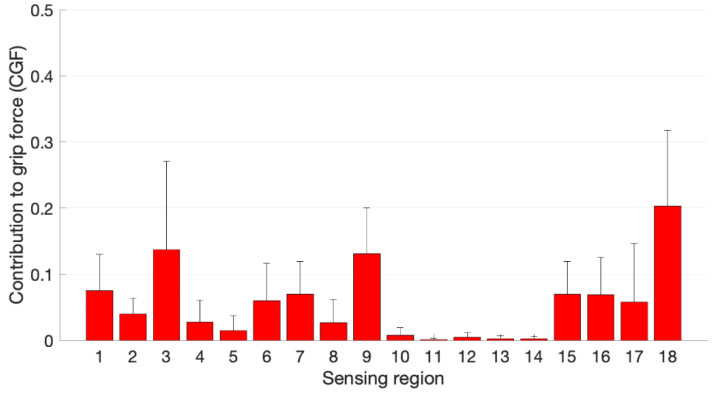
Contribution to grip force (CGF) for the different sensing regions (1–18) as average of the mean value for each task. The lines indicate the standard deviation.

Figure 9 shows a bar plot of the CGF in the eighteen regions obtained by averaging across the twenty-one different tasks. The sensing region 18, corresponding to the hypothenar eminence shows the highest contribution on average, 20.3%, followed by the distal phalanx of the index finger (region 3), with a contribution of 13.7%. The thumb contribution was on average close to 17.3%, distributed in regions 1 (distal, 7.5%), 2 (proximal, 4.0%) and 17 (thenar eminence, 5.8%). The lowest contributions came from sensing regions 11 to 14, corresponding to the proximal phalanx of the ring finger and the three phalanges of the little finger. However, the distal phalanx of the ring finger (region 9) presents a high CGF, 13.1%. The contribution of the middle finger is also important in both distal and intermediate phalanges, 6.0% and 7.0%, respectively. The distal palmar area is also contributing significantly on average, with a force sharing of 7.0% in region 15 and 6.9% in region 16. A higher standard deviation is observed in Figure 9 for some regions (3, 17, 18), indicating a greater variation in the contribution of these areas depending on the task performed.

**Figure 10:**
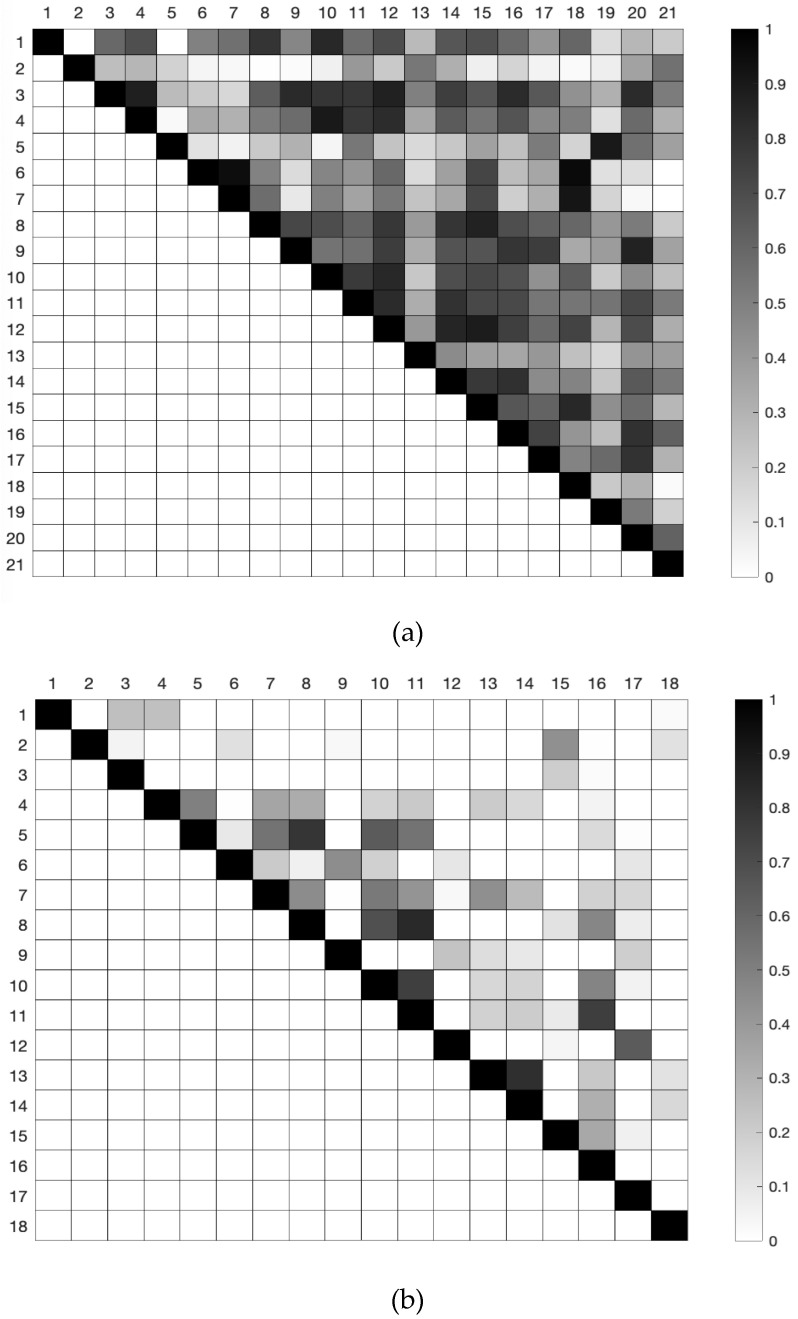
Linear correlation coefficient between tasks (**a**) and between regions (**b**), based on the CGF of the eighteen sensing regions.

Figure 10 shows a picture of the correlation among tasks (Figure 10a) and among regions (Figure 10b) based on the CGF results. The cell darkness indicates the Pearson correlation coefficient in a range from 0 to 1. A correlation coefficient above 0.7 is usually considered as a strong correlation. A correlation higher than 0.9 was obtained between tasks 5 (answering a phone call) and 19 (spraying insecticide) and also among tasks 6 (dialing phone numbers), 7 (typing on a computer keyboard), and 18 (cleaning a glass window with a cloth). This high correlation is also visible from the similar signature for these tasks in Figure 8. Correlation coefficients higher than 0.85 were also observed in Figure 10a for other pairs of tasks (3–4, 3–12, 4–10, 8–15, 9–20, 12–14, 12–15). We did not observe any substantial negative correlation between tasks. Some regions of the hand showed positive correlations of CGF throughout the different tasks (Figure 10b), mainly corresponding to combinations of proximal and medial phalanges of the fingers or with the distal palm region (5–8, 8–11, 10–11, 11–16, 13–14 pairs showed correlations above 0.75). A positive correlation was also observed between other regions, but to a lesser degree. An example is that between the distal thumb and the distal and middle phalanges of the index finger, which is expectable in pinch or lateral grasps. Some negative correlations in CGF were also observed between some of the regions (not visible with the scale used in Figure 10b). Of those, only three pairs of regions presented a correlation coefficient above 0.5 in magnitude, corresponding to pairs of regions between the distal phalange of the thumb or the index finger and the distal or medial phalanx of the middle finger (1–10, 3–6, 3–7).

Table 4 shows the maximum values of the peak force and the peak pressure for each sensing region during the experiments, with indication of the task in which they appeared. The highest peak force appeared in the palm (region 16) in task 2 (opening the door) and was close to 30 N. Other palm regions and fingers regions reached peak forces between 10 and 20 N for power tasks, such as task 21 (lifting the briefcase), tasks 17 or 19 (spraying), task 20 (ironing), or task 8 (opening a bottle). The highest peak pressure was close to 250 kPa and appeared in region 6 (distal middle finger) for task 17 (spraying). Peak pressures above 200 kPa appeared also in intermediate and proximal areas of the middle finger and in some palm regions.

Both “task” and “subject” factors were found to affect the GF significantly (*p* < 0.001) from the ANOVA tests. With respect to the CGF in the different regions, “task” and “subject” factors were also mostly significant. Table 5 shows the *p*-values for the regions not affected significantly by any of the factors, corresponding all to proximal or middle phalanges of the fingers or the thumb. The effect of subject’s sex on GF was found to be non-significant in the ANOVA tests for all the tasks, with *p*-values ranging between 0.16 and 0.98 among the different tasks. Its effect on CGF was also found non-significant from the MANOVA tests for all the tasks except for tasks 5 (*p* = 0.02) and 14 (*p* = 0.01).

## 4. Discussion

The dataset, methods, and results presented in this paper contribute to a better understanding of the tactile signatures of the human hand in a representative set of activities of daily living. The dataset contains more than one hundred million records of hand pressure in 361 hand locations for 21 different activities performed by 22 subjects. The paper also provides ideas to adequately conduct similar experiments with the commercial sensor employed and to analyze the data for distilling relevant information about how humans grasp and manipulate objects.

We made in this paper a partial analysis of the data in order to explain the main results, but further analyses are possible and can provide new clues about human grasping. We decided to group the results by sensing regions of the sensor, despite the data having a finer spatial resolution, in order to facilitate biomechanical explanations of the results and to make them more easily transferable to robotic or prosthetic applications.

Our results indicate that tactile signatures are significantly affected by the task and also by the subject. Only the contributions of the middle thumb and some other proximal and middle regions of the fingers seem to be less affected by the task or the subject. A significant effect of the task on the results was expected a priori, given the diversity of objects and forces involved in the different tasks. However, the results varied also depending on the subject, which could be more difficult to explain. Possible reasons for this are interpersonal differences, as a consequence of a different learning process, or a different adaptation of the hand size, or the hand strength to the objects or the tasks. However, anatomical differences also affected the fit of the glove and sensor to the hand, leading to contacts in different sensing areas from subject to subject. Given the characteristics of the sensor and the fact that its size is unique, it is clear that it will cover a lower fraction of the palm, or the fingers, in subjects with greater hands. Conversely, in the case of subjects with smaller hands the sensor regions can eventually contact laterally between them when flexing the fingers, introducing some distortion in the measurements, or even the saturation of some cells, as we observed. However, as can be seen in Figure 7, when the GF is scaled for a common mean GF, the differences among subjects do not prevent the recognition of a clear time course of the grip force during the task.

Overall, the Grip System from Tekscan Inc. worked consistently during the tests performed in this study and was easy to use, following the methodology described here. The use of the two thin plastic gloves over the cotton glove with the sensor, helped to protect the sensor and fixed its position with respect to the subject’s hand, not affecting importantly the execution of the tasks. Compared to the STAG sensor proposed in [14], the Grip System has a lower number of sensels, 361 versus 548. The spatial resolution in the fingers is comparable but the Grip System does not cover the palm as well as the STAG does. Additionally, STAG seems to exhibit greater flexibility and compliance. The Grip System has the advantage of being commercially available and thus having a faster setup but at a higher cost.

The custom method used here for time warping the data from different subjects performing the same task worked efficiently to catch the important events in the time series and is applicable to synchronizing different trials of grasping experiments with differences in the speed of execution or in the trigger events. We observed a better behavior of this method as compared to the use of the Matlab built-in function “dtw” because it only scales time in three segments of the time scale (initial, middle, and final), avoiding the artificial repetition of measured signals that appears in traditional dynamic time warping [30].

Our results show that the grasping forces necessary for simple everyday activities are quite different, ranging from less than 5–10 N to about 100 N in the tasks considered here. The peak forces shown in Table 4 for each hand region can be considered as desirable design targets for anthropomorphic artificial hands for service robots. The values in Table 4 can also be useful to estimate the torque requirements for the actuators of the artificial fingers and as a reference for their mechanical design, which has to be strong enough to withstand these forces and pressures. However, the limitations of our study must be taken into account to consider some safety factors on the use of these values, because they were obtained from a limited set of objects and tasks. Actual forces necessary with other objects or for other tasks may be higher. Factors, such as friction coefficient, force required by the task, object geometry, and available contact area, can lead to higher force or pressure requirements in the whole hand or in specific hand regions.

The analysis of the contribution of the different hand regions to the grip force highlights the main areas contributing to the grasping force in the ADL studied and provide valuable information for the design of robotic or prosthetic hands. Simplified tactile signatures, such as those shown in Figure 8, can be helpful for controlling artificial hands in similar activities or for training machine learning models to this end. We found some correlations between tactile signatures of different tasks. The analysis of these correlations for a higher number of activities can help to identify the most important force patterns that artificial hands must be able to reproduce. The hypothenar eminence region appeared to be the region with the highest contribution, but this result could have been affected by the fact that this area is sometimes acting as a support of the hand, which is in contact with the table in activities such as writing or using keyboards. Distal finger regions are loaded on average more than middle or proximal regions, with the distal index being the highest contributor of the fingers, because of its main role in pinching or pressing activities. The higher positive correlations observed among middle and proximal fingers regions with respect to those between distal fingers indicate that they act less independently. This result agrees with the differences observed in neural control for power and precision tasks [31]. The higher independence between distal finger regions is necessary for dexterous control in precision tasks, such as brushing teeth or writing with a pen, while the higher coordination in proximal regions is attributed to their necessary collaboration for producing higher forces in power tasks, such as ironing, opening a door, or lifting a briefcase. Designers of anthropomorphic artificial hands could consider this coordination or independence between hand regions for improving the actuation or control of the hands.

A limitation of our work is that we only collected pressure information on the right hand, for right-handed people. Despite the execution of most of the tasks involved only the right hand, the left hand also collaborated in some of them, such as 7 (typing on a computer keyboard), 8 (opening a bottle and pouring water), 11 (cutting play-dough in a plate with a knife), and 15 (serving toothpaste in a toothbrush). Some comments can be made about the expected profiles for the left hand in those tasks. In task 7, we expect results for the left hand similar to those of the right hand in the same task, given the symmetry of the task. For task 8, the left hand is grasping the bottle while the right hand is opening the cap; a pressure distribution in the left hand similar to that observed for the right hand in task 9 (drinking water from a glass) could be expected. In task 11, the left hand is grasping a fork to restrain the motion of the play-dough and hence its pressure distribution could be similar to that observed in task 12 (pricking with a fork) for the right hand. In task 15, the left hand grasps the tube of toothpaste first and the toothbrush later. Estimations of the pressure distribution and forces for this left hand can be done using the recorded information for the right hand while grasping the same objects at other moments of the same task or in task 14 (brushing teeth).

## Figures and Tables

**Table 1:** Task description grouped by setup used (see Figure 1), with indication of objects manipulated and subject’s posture.

Task	Description	Setup	Objects Manipulated with Right Hand	Subject’s Posture
1	Opening a lock with a key	1	key	*Standing*
2	Turning a handle to open a door	1	door handle	*Standing*
3	Handling a book	2	book	*Standing*
4	Writing with a pen	2	pen	*Seated*
5	Asking a phone call with a table phone	2	phone handset	*Seated*
6	Dialing phone numbers	2	phone keys	*Seated*
7	Typing on a computer keyboard	2	keyboard keys	*Seated*
8	Opening a bottle and pouring water	3	bottle, bottle cup	*Seated*
9	Drinking water from a glass	3	glass	*Seated*
10	Eating soup with a spoon	3	spoon	*Seated*
11	Cutting play-dough in a plate with a knife	3	knife	*Seated*
12	Pricking play-dough portions from a plate with a fork	3	fork	*Seated*
13	Opening and closing a water tap	4	water tap	*Standing*
14	Brushing teeth	4	toothbrush	*Standing*
15	Serving toothpaste in a toothbrush	4	toothpaste tube, tube cap	*Standing*
16	Taking a comb from a drawer and combing hair	4	drawer handle, comb	*Standing*
17	Spraying with a pistol grip cleaner on a glass window	5	pistol grip sprayer	*Standing*
18	Cleaning a glass window with a cloth	5	cleaning cloth	*Standing*
19	Spraying insecticide	5	aerosol can	*Standing*
20	Ironing clothes	6	iron	*Standing*
21	Lifting a briefcase from ground level	6	briefcase	*Standing*

**Table 2:** Sequence of actions performed by the subject in each task.

Task	Sequence of Actions
1	Taking a key from the table; inserting it in the lock; twisting it 180° clockwise; twisting it 180° counter-clockwise; extracting the key from the lock; leaving the key on the table
2	Grasping the handle; twisting it completely to open; opening the door; closing the door; returning the handle to the initial position and releasing it
3	Grasping a book from a shelf; moving the book to the table; leaving the book on the table with the cover facing up
4	Grasping the pen from the table; writing the text “*EN UN LUGAR DE LA MANCHA DE CUYO NOMBRE NO QUIERO ACORDARME*” in the paper; leaving the pen on the table
5	Picking up the phone with the right hand; moving the handset to the right ear; waiting for about three seconds; hanging up the phone
6	Dialing the sequence of numbers 917,352 by pressing the phone keys with the index finger
7	Using the keyboard with both hands to write the text “*EN UN LUGAR DE LA MANCHA DE CUYO NOMBRE NO QUIERO ACORDARME*”
8	Grasping the bottle with the left hand; opening the cap with the right hand; leaving the cap on the table; pouring water in a glass with the right hand to fill half of the glass; leaving the bottle on the table
9	Grasping the glass filled of water from the table; drinking glass content; leaving the glass on the table
10	Grasping the spoon from the table; simulating taking three tablespoons; leaving the spoon in the plate
11	Grasping the knife with the right hand and the fork with the left hand from the table; cutting three pieces of play-dough; leaving the knife and fork in the plate
12	Grasping the fork; pricking a piece of clay and moving it to the mouth to simulate eating (three times); leaving the fork in the plate
13	Opening the tab for two seconds; closing the tab; releasing the tab
14	Grasping the toothbrush from a glass; brushing the teeth for five seconds; leaving the toothbrush in the glass
15	Grasping the tube of toothpaste with the right hand; switching the tube to the left hand; unscrewing the cap with the right hand; switching the tube back to the right hand; grasping the toothbrush with the left hand; serving the toothpaste; leaving the tube
16	Opening a drawer with the right hand; grasping a comb from the drawer; combing for five seconds; leaving the comb; closing the drawer
17	Grasping glass cleaner pistol from the table; spraying three times on the vertical glass; leaving the glass cleaner pistol on the table
18	Grasping the cloth; cleaning the glass; leaving the cloth
19	Grasping the insecticide; spraying for half a second; leaving insecticide
20	Grasping the iron from the table; ironing for five seconds; leaving the iron in the table
21	Grasping the briefcase from the ground; lifting it to the table level; releasing it lying down on the table

**Table 3:** Sensing regions of the sensor and corresponding anatomical locations.

Sensing Region	Approximate Anatomic Location	Number of Sensels
1	Thumb, distal phalanx	16
2	Thumb, proximal phalanx	12
3	Index finger, distal phalanx	16
4	Index finger, intermediate phalanx	12
5	Index finger, proximal phalanx	12
6	Middle finger, distal phalanx	16
7	Middle finger, intermediate phalanx	12
8	Middle finger, proximal phalanx	12
9	Ring finger, distal phalanx	16
10	Ring finger, intermediate phalanx	12
11	Ring finger, proximal phalanx	12
12	Little finger, distal phalanx	16
13	Little finger, intermediate phalanx	12
14	Little finger, proximal phalanx	12
15	Distal palm, second metacarpal	16
16	Distal palm, third to fifth metacarpal	60
17	Thenar eminence	45
18	Hypothenar eminence	52

**Table 4:** Maximum peak force and peak pressure in the sensing regions, averaged among subjects, with indication of the task in which they appeared.

Sensing Region	Peak Force (N)	Task	Peak Pressure (kPa)	Task
1	10.2	8	181.5	8
2	5.8	8	153.6	13
3	7.8	8	153.5	11
4	8.3	8	128.9	2
5	12.0	21	183.3	21
6	13.2	17	268.3	17
7	14.6	21	214.9	21
8	11.1	2	201.4	2
9	18.2	20	170.6	20
10	7.0	21	134.3	21
11	2.5	2	114.1	2
12	1.4	19	78.4	19
13	0.6	12	30.9	2
14	0.6	2	45.6	2
15	11.8	2	193.4	2
16	28.1	2	237.9	2
17	17.6	19	126.3	19
18	18.4	2	200.4	21

**Table 5:** Significance levels (*p*-value) from the ANOVA tests on CGF with factors “subject” and “task” for regions showing non-significant effect from any of the two factors (significant effect with *p* < 0.05 for regions not shown).

Sensing Region	Task (*p*-Value)	Subject (*p*-Value)
2	0.176	0.000
4	0.000	0.205
5	0.000	0.275
10	0.000	0.078
14	0.063	0.000

## Data Availability

The dataset presented in this study is openly available in Zenodo at https://doi.org/10.5281/zenodo.4607289 (accessed on 7 April 2021) [29].

## Abbreviations

ADL	Activities of daily living
CGF	Contribution to grip force
DTW	Dynamic Time Warping
ICF	International Classification of Functioning, Disability and Health
GF	Grip force

## References

[1] Khurshid A., Ghafoor A., Afzaal M. (2015). Robotic Grasping and Fine Manipulation Using Soft Fingertip. Adv. Mechatron..

[2] Bezak P., Bozek P., Nikitin Y. (2014). Advanced robotic grasping system using deep learning. Procedia Eng..

[3] Ozawa R., Tahara K. (2017). Grasp and dexterous manipulation of multi-fingered robotic hands: A review from a control view point. Adv. Robot..

[4] Du G., Wang K., Lian S., Zhao K. (2020). Vision-Based Robotic Grasping from Object Localization, Object Pose Estimation to Grasp Estimation for Parallel Grippers: A Review.

[5] Saxena A., Driemeyer J., Kearns J., Ng A.Y. (2007). Robotic grasping of novel objects. Adv. Neural Inf. Process. Syst..

[6] Li Y., Lei Q., Cheng C., Zhang G., Wang W., Xu Z. A review: Machine learning on robotic grasping. Proceedings of the Eleventh International Conference on Machine Vision (ICMV 2018).

[7] Levine S., Pastor P., Krizhevsky A., Ibarz J., Quillen D. (2018). Learning hand-eye coordination for robotic grasping with deep learning and large-scale data collection. Int. J. Robot. Res..

[8] Yang Y., Liang H., Choi C. (2020). A Deep Learning Approach to Grasping the Invisible. IEEE Robot. Autom. Lett..

[9] Li M., Bekiroglu Y., Kragic D., Billard A. Learning of grasp adaptation through experience and tactile sensing. Proceedings of the IEEE International Conference on Intelligent Robots and Systems.

[10] Dang H., Allen P.K. (2014). Stable grasping under pose uncertainty using tactile feedback. Auton. Robot..

[11] Hyttinen E., Kragic D., Detry R. Learning the tactile signatures of prototypical object parts for robust part-based grasping of novel objects. Proceedings of the IEEE International Conference on Robotics and Automation.

[12] Wang Q. (2019). Improving Tactile Sensing for Robotic Grasping.

[13] Hogan F.R., Bauza M., Canal O., Donlon E., Rodriguez A. Tactile Regrasp: Grasp Adjustments via Simulated Tactile Transformations. Proceedings of the 2018 IEEE/RSJ International Conference on Intelligent Robots and Systems (IROS).

[14] Sundaram S., Kellnhofer P., Li Y., Zhu J.Y., Torralba A., Matusik W. (2019). Learning the signatures of the human grasp using a scalable tactile glove. Nature.

[15] Aoki T., Niu X., Latash M.L., Zatsiorsky V.M. (2006). Effects of friction at the digit-object interface on the digit forces in multi-finger prehension. Exp. Brain Res..

[16] Santello M., Soechting J.F. (2000). Force synergies for multifingered grasping. Exp. Brain Res..

[17] Kuo L.-C., Chen S.-W., Lin C.-J., Lin W.-J., Lin S.-C., Su F.-C. (2013). The force synergy of human digits in static and dynamic cylindrical grasps. PLoS ONE.

[18] Lee S.-J.S., Kong Y.Y.-K., Lowe B.B.D., Song S. (2009). Handle grip span for optimising finger-specific force capability as a function of hand size. Ergonomics.

[19] McGorry R.W., Lin J.-H. (2007). Power grip strength as a function of tool handle orientation and location. Ergonomics.

[20] Kong Y.-K., Seo M.-T., Kang H.-S. (2014). Evaluation of total grip strength and individual finger forces on opposing (A-type) handles among Koreans. Ergonomics.

[21] Nicholas J.W., Corvese R.J., Woolley C., Armstrong T.J. (2012). Quantification of hand grasp force using a pressure mapping system. Work.

[22] Pataky T.C., Slota G.P., Latash M.L., Zatsiorsky V.M. (2012). Radial force distribution changes associated with tangential force production in cylindrical grasping, and the importance of anatomical registration. J. Biomech..

[23] Wu J.Z., Dong R.G., Warren C.M., Welcome D.E., McDowell T.W. (2014). Analysis of the effects of surface stiffness on the contact interaction between a finger and a cylindrical handle using a three-dimensional hybrid model. Med. Eng. Phys..

[24] Manis R.P., Santos V.J. (2015). Independent digit contributions to rotational manipulation in a three-digit pouring task requiring dynamic stability. Exp. Brain Res..

[25] Pylatiuk C., Kargov A., Schulz S., Döderlein L. (2006). Distribution of grip force in three different functional prehension patterns. J. Med. Eng. Technol..

[26] Hermsdörfer J., Li Y., Randerath J., Goldenberg G., Eidenmüller S., Eidenmuller S. (2011). Anticipatory scaling of grip forces when lifting objects of everyday life. Exp. Brain Res..

[27] Cepriá-Bernal J., Pérez-González A., Mora M.C., Sancho-Bru J.L. (2017). Grip force and force sharing in two different manipulation tasks with bottles. Ergonomics.

[28] Silva D.C., Paschoarelli L.C., Medola F.O. (2019). Evaluation of two wheelchair hand rim models: Contact pressure distribution in straight line and curve trajectories. Ergonomics.

[29] Pérez-González A., Cepria-Bernal J. (2021). Dataset of tactile signatures of the human hand in twenty one activities of daily living. Zenodo.

[30] Folgado D., Barandas M., Matias R., Martins R., Carvalho M., Gamboa H. (2018). Time Alignment Measurement for Time Series. Pattern Recognit..

[31] Dietz V. (2020). Neural coordination of bilateral power and precision finger movements. Eur. J. Neurosci..

